# Reviewed article: Brito ES, Texeira MA, Martins RS, Pinheiro BHG, Rocha ACM, Oliveira CN, Paula TF, Dornelles TM. HIV prevalence and factors associated with HIV positivity among Black people in primary care in Porto Alegre, Brazil, 2020-2022: a cross-sectional study. Epidemiol Serv Saude. 2025:34;e20240014

**DOI:** 10.1590/S2237-96222025v34e20240014.a

**Published:** 2025-05-12

**Authors:** Tayanny Margarida Menezes Almeida Biase

**Affiliations:** Universidade Estadual de Campinas, School Pharmaceutical Science

**Table:** 

Rating	Excellent	Good	Average	Below average	Poor
Interest		✓			
Quality			✓		
Originality			✓		
Overall		✓			

**Table:** 

Questionnaire	Yes	No	Not applicable
Does the manuscript contain new and significant information to justify publication?	✓		
Does the Abstract (Summary) clearly and accurately describe the content of the article?	✓		
Is the problem significant and concisely stated?	✓		
Are the methods described comprehensively?	✓		
Are the interpretations and conclusions justified by the results?	✓		
Is adequate reference made to other work in the field?	✓		

## Manuscript Structure:

Length of article is: Adequate

Number of tables is: Adequate

Number of figures is: Adequate

Please state any conflict(s) of interest that you have in relation to the review of this paper (state “none” if this is not applicable): None

## Comments

O trabalho demonstra relevância e possui metodologia robusta. O texto apresenta linguagem clara e concisa. Os parágrafos foram bem desenvolvidos e a informação principal é apresentada. Sugiro as seguintes considerações:

Defina cada variável, por exemplo, idade (categorizada em anos: 12 a 18, 18 a 34, etc; caso de tuberculose: sim e não).

Sugiro revisão do texto e padronização para citar valores numéricos.

Sugiro incluir nos métodos uma frase indicando que o check-list Strobe para estudos transversais foi seguido.

O terceiro parágrafo da introdução traz a referência de número seis Giannouchos (2023) não é coerente, os valores de incidência informados estão na introdução desse trabalho e não fazem parte dos resultados. Sugiro aos autores procurar a fonte primária dos dados. Também sugiro revisar todas as referências e observar a compatibilidade dos dados.

Sugiro que para cada tabela apresentada seja incluído ao final da legenda o número de participantes incluídos na análise para facilitar a compreensão. 

